# Positional error and time-activity patterns in near-highway proximity studies: an exposure misclassification analysis

**DOI:** 10.1186/1476-069X-12-75

**Published:** 2013-09-08

**Authors:** Kevin J Lane, Madeleine Kangsen Scammell, Jonathan I Levy, Christina H Fuller, Ron Parambi, Wig Zamore, Mkaya Mwamburi, Doug Brugge

**Affiliations:** 1 Boston University School of Public Health, Boston, MA, USA; 2 Georgia State University Institute of Public Health, Atlanta, GA, USA; 3 Department of Radiation Oncology, Mass General Hospital, Boston, MA, USA; 4 Somerville Transportation Equity Partnership, Somerville, MA, USA; 5 Tufts University School of Medicine, Boston, MA, USA

**Keywords:** Highway proximity, Time-activity, Traffic, Geocoding, Exposure misclassification

## Abstract

**Background:**

The growing interest in research on the health effects of near-highway air pollutants requires an assessment of potential sources of error in exposure assignment techniques that rely on residential proximity to roadways.

**Methods:**

We compared the amount of positional error in the geocoding process for three different data sources (parcels, TIGER and StreetMap USA) to a “gold standard” residential geocoding process that used ortho-photos, large multi-building parcel layouts or large multi-unit building floor plans. The potential effect of positional error for each geocoding method was assessed as part of a proximity to highway epidemiological study in the Boston area, using all participants with complete address information (N = 703). Hourly time-activity data for the most recent workday/weekday and non-workday/weekend were collected to examine time spent in five different micro-environments (inside of home, outside of home, school/work, travel on highway, and other). Analysis included examination of whether time-activity patterns were differentially distributed either by proximity to highway or across demographic groups.

**Results:**

Median positional error was significantly higher in street network geocoding (StreetMap USA = 23 m; TIGER = 22 m) than parcel geocoding (8 m). When restricted to multi-building parcels and large multi-unit building parcels, all three geocoding methods had substantial positional error (parcels = 24 m; StreetMap USA = 28 m; TIGER = 37 m). Street network geocoding also differentially introduced greater amounts of positional error in the proximity to highway study in the 0–50 m proximity category. Time spent inside home on workdays/weekdays differed significantly by demographic variables (age, employment status, educational attainment, income and race). Time-activity patterns were also significantly different when stratified by proximity to highway, with those participants residing in the 0–50 m proximity category reporting significantly more time in the school/work micro-environment on workdays/weekdays than all other distance groups.

**Conclusions:**

These findings indicate the potential for both differential and non-differential exposure misclassification due to geocoding error and time-activity patterns in studies of highway proximity. We also propose a multi-stage manual correction process to minimize positional error. Additional research is needed in other populations and geographic settings.

## Background

People residing in close proximity to highways and freeways are exposed to higher concentrations of potentially harmful pollutants such as ultrafine particulate matter (UFP; aerodynamic diameter <100 nm), black carbon (BC), nitrogen oxides (NOx), and carbon monoxide (CO). Peak exposures to traffic-related pollutants occur during travel on roads where in-cabin time-activity significantly contributes to an individual’s total personal exposure profile [1-3]. Concentrations of these pollutants exponentially decay with increasing distance from the highway, with highest concentrations appearing over the first 50 meters and distribution observed up to 400–500 m [4-8]. The characterization of UFP and other near-road exposures is complicated by observed diurnal and seasonal changes in concentrations, with high concentrations in early morning and winter seasons [4,8]. Temporal variability of UFP concentrations has been shown to follow morning rush hour patterns, with increases in UFP concentrations between a factor of 2 and 5 observed during local rush hour periods (6:30–8:00 AM) [4,5,7-9]. The spatial and temporal variability seen in near-highway air pollutants requires precise and innovative methods to assess exposure in epidemiological studies.

Two potentially significant sources of exposure error in near-roadway epidemiology are the time-activity patterns of populations, including time away from home, and the geographic accuracy of locating the residential position [10-12]. Traffic-related air pollutant studies have often relied upon residential distance to highway and major roadways as an indicator of exposure as well as modeled concentrations of fine particulate matter (PM_2.5_), NOx, UFP and other pollutants [13-16]. However, many such studies do not account for variable time-activity patterns, which may influence epidemiological study findings through misclassification of exposure [11,17,18]. Additionally, the reliance upon street-network geocoding of residential addresses has been shown to introduce positional error with only a small number of articles using parcel or ortho-photo correction methods [10,19-21]. These studies suggest the need for further investigation into the effects of positional error on exposure misclassification in health studies.

In this study we examine the influence of time-activity patterns and geocoding error on exposure misclassification in a study of exposure to pollution from highways. We consider the nature of the exposure error and whether time-activity patterns or geocoding errors are differentially distributed across the population. We also propose a multi-stage manual correction process to minimize positional error.

## Methods

### Study population

**Figure 1:**
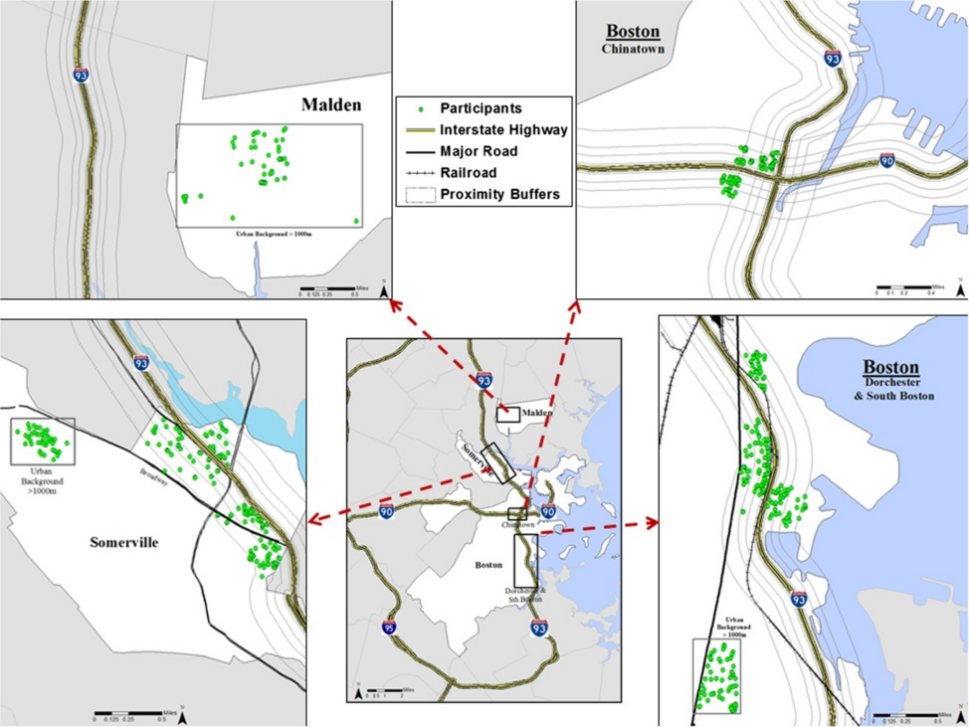
**CAFEH participants ortho**-**photo corrected residences by study area and distance to highway buffer groupings (N=703).**

The Community Assessment of Freeway Exposure and Health study (CAFEH) is a community-based participatory research (CBPR) study of near-highway traffic-related air pollutants and cardiovascular health in individuals 40+ years of age living within close proximity to major highways [22]. The CAFEH sample was established via a geographically-weighted randomly-selected address recruitment effort, plus a convenience sample within each study area in the cities of Boston, Malden and Somerville MA from July 2009 through December 2012 (Figure 1). Within the City of Boston, the CAFEH study recruited participants from three neighborhoods - Chinatown, Dorchester and South Boston - that contain different geographic and demographic profiles. Interstate highway (I-93) bisects Somerville and serves as a neighborhood boundary for South Boston and Chinatown while the recruitment area for Malden is located greater than 1000 m away from the nearest highway (Figure 1). A second interstate highway (I-90) is an open sunken highway that runs perpendicular to I-93 and intersects the Chinatown neighborhood. Northeast of the Chinatown neighborhood there is a tunnel exit for I-93 that is approximately 225 m from a portion of the study participants residing in this study area. Convenience and randomly selected participants are spread throughout all of the study areas regardless of distance to highway.

Participants completed an in-home administered questionnaire (N = 703) providing demographic information (age, gender, income, education, race, etc.) and information on a variety of other topics related to our exposure and health outcomes of interest (diet, physical activity, stress, medications, diagnosed illnesses, etc.). Participants who completed an in-home questionnaire were asked to also come to one or two clinic visits with a study nurse to measure blood pressure and draw a blood sample (first clinic N = 454; second clinic N = 222).

### Geographic data

ESRI ArcGIS 10.1 (ESRI, Redlands, CA) was used for all geocoding and spatial data processing. Residential street addresses and apartment numbers (when applicable) were verified by the CAFEH field team during in-home interviews. The study sample consisted of 703 participants with verified addresses, not including one individual with an incomplete street address. Residential addresses were geocoded using three separate address location datasets that utilized an internal geocoding service: StreetMap USA street network (StreetMap) from 2010, the TIGER street network from 2011, and parcel geo-databases. Parcel address datasets were obtained for Somerville and Boston from the GIS and city planning departments in 2011 and 2012 respectively. Malden parcel data and ortho-photos for all three study cities were obtained from the Massachusetts Office of Geographic Information (MassGIS) for 2012.

Address matching is based upon accuracy of the datasets matched on spelling between the address dataset and the geocoding service. Street network geocoding uses a dual address range with interpolation to assign an address point to left and right sides of the road based on start and end numbers for road segments. The dual address range allows for greater match-rates to occur since they are matched on the spelling of the street name data fields. The geocoding process then identifies which road segment contains the address number within the range without confirming its existence [23]. Parcel geocoding relies upon 1:1 address matching to individual land polygons or their centroids which contain a singular address number that can be matched. The dual address range interpolation process of street-network geocoding improves the likelihood of an exact match compared to parcel geocoding, but parcels are considered to have higher spatial accuracy [23]. A spelling sensitivity score of 80 with a minimum match score of 60 was used during geocoding for TIGER, StreetMap and parcel matching. Manual interactive matching was performed for addresses that were either unmatched or had a score below 80 to obtain the highest number of reliable matches. StreetMap and TIGER geocoding for all complete participant addresses resulted in a 99% match with less than 4% of addresses that did not report a match score of 100. Parcel address geocoding matched 92% of all addresses. The positional error analysis was restricted to the 647 participant addresses that were successfully geocoded to StreetMap, TIGER and parcel databases.

**Figure 2:**
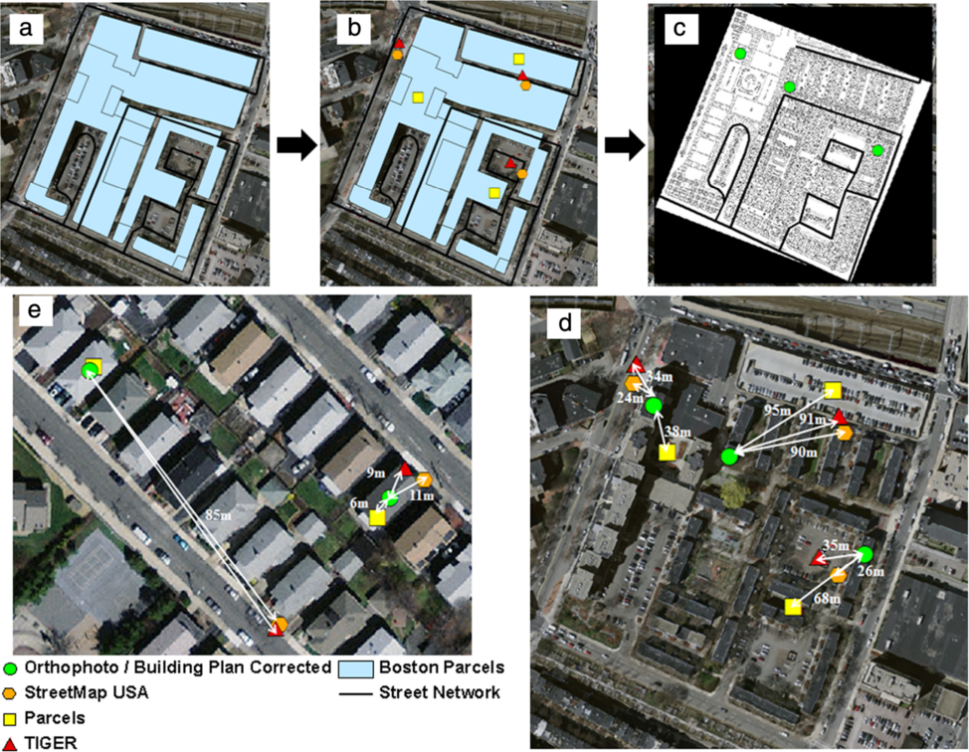
**Example for ascertaining true ground location and determining positional error for large multi**-**building parcels and single/multi-family home (i.e. duplexes and triple**-**deckers). a**. Example of a large multi-building parcel with both senior and affordable housing units comprised of 15 different parcels, **b**. Three different addresses have been geocoded to both street-networks (red triangles & orange hexagons) and parcels (yellow squares), **c**. Building location with unit layouts are georeferenced in ArcGIS using ortho-photos to ascertain true location (green circles), **d**. Distance between Street-network, parcel and the corresponding true ground location determined through building and unit master plans for large multi-building parcels, **e**. Distance between Street-network, parcel and the corresponding ortho-photo corrected to the middle of the home for large multi-building parcels.

We established true ground location by manually moving all parcel geocoded address points from the middle of the parcel (centroid) to the middle of the residential building using ortho-photos (Figure 2). Ortho-photos are vertical aerial photographic images of the Earth that have been geo-referenced to known GPS coordinates and geometrically transformed to account for topographic relief through a rectification process [24]. The ortho-photo images used for this analysis came from 2008–2009 flyovers of Massachusetts and are listed as having a horizontal error of less than one meter [25]. Ortho-photos are considered to have more reliable spatial accuracy than street networks and parcels since ortho-photos are used to create vector maps through a process known as digitizing in GIS. Distance measures obtained from ortho-photo maps are considered more reliable than digitized vector maps. Ortho-photos are considered a gold standard for location verification and have been used in studies to assess positional error in street network geocoding [19,21,26].

Parcel building and apartment layout maps were obtained through community partners contacting building management and housing authorities, and these maps were geo-referenced within ArcGIS to the ortho-photos (Figure 2). This allowed for correct building and unit assignment for participants residing on large multi-building parcels and in apartment buildings, such as public and senior housing facilities. These plans were used for building and apartment unit assignment for 27% of the study population. Large multi-unit buildings and multi-building parcels that did not have floor plans or building layouts had their parcel geocoded positions corrected to the middle of the building or parcel (n = 50, 7% of total study population). It should be noted that geo-referenced scanned parcel/building layout maps still have the potential for errors in spatial accuracy such as the stretching of an image between two or more geo-referenced positions, also referred to as “rubber sheeting”. Latitude and longitude coordinates in the Massachusetts state plane projection were obtained for StreetMap, TIGER, parcel and ortho-photo corrected residential positions and were used to calculate the distance between the three geocoding methods and the ortho-photo corrected position.

The Massachusetts Department of Transportation road centerline layer contains a road width field that we used in ArcGIS to convert the centerlines into an edge of highway buffered road layer that we inspected for accuracy to the ortho-photos and used to calculate Euclidean distance to the edge of the nearest highway. The edge of highway buffered road layer created from the road width field covers only the vehicle traveling lanes while excluding breakdown/emergency lane or the shoulder of the road. Distance to the nearest highway was determined for each geocoding method and the ortho-photo corrected residential locations to analyze the potential for exposure misclassification in a near-highway distance proximity study.

### Micro-environment time-activity

**Figure 3:**
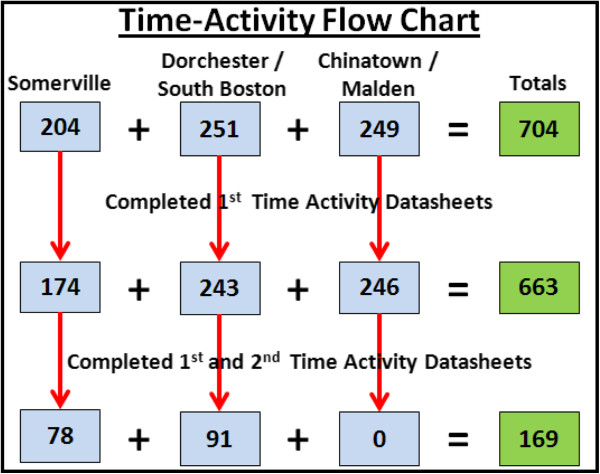
**Micro**-**environment time**-**activity participant completion flow chart by study area.**

Hourly time-activity data were collected during the in-home survey and as part of the second clinic visit via questionnaire for the most recent workday and non-workday for participants who worked full- or part- time, or were full-time students. Participants who were retired, disabled, worked in the home or were unemployed were asked to provide information on their most recent weekday and weekend. Workday and weekday data were pooled together along with non-workday and weekend for analysis except when stratified by employment status. Based on this method, hourly micro-environment data were recorded for time spent inside of the home, outside of the home (in open air), at work/school, and at “other locations” (which include non-highway travel, open air, and indoor). Participants were asked to report highway travel separately on a minute scale for each hour of the day without detail on mode of transport. Highway travel data allowed for fractional integration with hourly micro-environment data. For example, a participant could indicate that they were inside the home micro-environment from 6–7 AM on a workday, but also report 30 minutes of highway travel during the same hour. This would allow for 30 minute contributions to both the inside of home and highway micro-environments during the 6–7 AM hour. Micro-environment time-activity analysis was restricted to participants with a complete 24-hour record for both the workday and non-workday questionnaire (N = 663) (Figure 3).

CAFEH participants recruited during the first two years from the Somerville, Dorchester and South Boston study areas were asked to take part in two clinics attended by 222 study participants, with 169 participants fully completing a second time-activity datasheet for both workday/weekday and non-workday/weekend (for a total of four time-activity datasheets) (Figure 3). There was an average of 5.4 months between in-home surveys and second clinic surveys, capturing seasonal variation. Data from participants who completed time-activity datasheets as part of the in-home survey and second clinic were examined for within-person variability of micro-environment time allocation.

### Statistical analysis

All statistical analyses were performed using SAS® (Statistical Analysis Software, Cary, North Carolina) version 9.12. Bivariate analyses were conducted using t-tests and Wilcoxon tests to compare means and medians for normally and non-normally distributed continuous variables, respectively. Analysis of variance (ANOVA), with a post-hoc Tukey test, was used to compare means of normally distributed continuous variables between multiple category variables. Differences in medians for non-normally distributed continuous variables for multiple groups were calculated using Wilcoxon tests with a post-hoc Bonferroni correction for multiple comparisons (α/# of tests). Chi-square analysis was used to compare differences in proportions. All statistical tests were two-sided and a p-value <0.05 was considered statistically significant.

Paired t-tests were conducted to examine the mean difference in total hours spent in each micro-environment between the first and second survey. Tests of mean difference betw the first and second survey were restricted to only those participants that had fully completed all four micro-environment time-activity datasheets (N = 169) obtained during the in-home questionnaire and second study clinic. Multi-variable regression models were used to explain the variability of time spent inside of home for those who completed a second questionnaire as a function of demographic and other variables.

We examined predictors of total and hourly time allocation for the “inside home” micro-environment using univariate regression models. A multivariate regression model was run to examine the amount of variation that could be explained for the daily total “inside home” micro-environment as a function of potential confounders or effect modifiers that most epidemiological studies would have collected (i.e. age, gender, income, education, race and employment status). A logistic regression model using the same predictors was run to examine the odds ratio of participants reporting being inside of the home for each hour of the day.

Analysis of spatial error in geocoding methodologies consisted of descriptive statistics and comparison of ortho-photo corrected locations to each geocoding methodology for measures of agreement as part of a proximity to highway exposure study. For this analysis, we presumed that study participants were assigned to one of six distance to highway categories (0–50 m, 51–150 m, 151–250 m, 251–450 m, 451–999 m and > =1000 m), and we evaluated the ability of StreetMap, TIGER and parcel geocoding to correctly assign participants to their true distance bins.

## Results

### Geocoding positional error

Ortho-photo corrected residential locations were compared to StreetMap, TIGER, and parcel residential locations (Table 1). As observed elsewhere [27] the distribution of positional errors was highly skewed with a small number of extreme values, so we focused on median positional error in our core comparisons. StreetMap and TIGER street network geocoding were found to have a significantly greater median positional error (23 m and 22 m respectively) than parcel geocoding (8 m). Street network geocoding also had a significantly higher median positional error than parcel geocoding when stratified by study area, with the exception of the South Boston neighborhood which included only 14 addresses in one large public housing complex (Additional file 1: Table S1).

**Table 1:** **Distance in meters between each geocoding method and ortho**-**photo corrected residential position by housing type**

	**Parcels**	**StreetMap USA**	**Tiger**
**All participants (N = 647)**			
Mean (SD)	21.48 (78)**	38.72 (90.07)*	48.94 (203)*
Median	7.5**	22.62*	21.81*
90^th^ Percentile	42.42	68.24	74.6
95^th^ Percentile	65.09	83.35	95.73
Min - Max	0 – 1352	0.8 – 1289	2.3 – 4453
**Housing type**			
**Large multi**-**unit parcels and buildings (N = 196)**			
Mean (SD)	37.57 (47.9)	40.51 (27.6)	51.05 (93.6)
Median	24.25* ^y^	27.61* ^y^	36.56** ^y^
90^th^ Percentile	112.09	81.63	91.43
95^th^ Percentile	85.9	90.39	104.07
Min - Max	0.37 – 406.59	4.59 – 168.62	5.69 – 1280.44
**Other housing (N = 451)**			
Mean (SD)	14.49 (86.8)** ^y^	37.94 (106.4)*	48.03 (235.3)*
Median	3.82** ^y^	19.8* ^y^	18.71* ^y^
90^th^ Percentile	21.02	58.54	78.41
95^th^ Percentile	30.66	74.27	60.14
Min - Max	0 – 1352.34	0.8 – 1289	2.3 – 4453.29

The analysis omits those addresses not successfully geocoded to both the TIGER and Parcel datasets (n = 647).

* Indicates a significant (P<0.05) difference between one other geocoding method.

** Indicates a significant (P<0.05) difference between two other geocoding methods.

^y^ Indicates a significant (P<0.05) difference between housing type within the same geocoding method.

To better understand the effect of housing type on positional error, we compared large multi-building and multi-unit parcels with “other” housing stock (defined as single family as well as double and triple-decker homes). The median positional error for large multi-building and multi-unit parcels (24 m) was more than five times the median positional error for single and multi-family homes (3.8 m). Parcel geocoding had a significantly lower median positional error compared to both street network geocoding datasets for the “other” housing stock (Table 1). No appreciable difference in positional error was detected between the street network geocoding methods and parcel geocoding when analysis was restricted to large multi-building and multi-unit parcels.

We examined the association between positional error and the demographic variables age, education, employment, gender, income, and race using univariate regression analysis. None of these demographic variables were found to be significant predictors of positional error for any of the geocoding methods (results not shown).

Using a distance to highway categorical variable in the context of a highway proximity study, both street network geocoding datasets had more errors than parcel geocoding, with fewer confirmed matches and higher percent false positives and false negatives in all distance groupings (Table 2). The probability of the street networks correctly assigning participants to their true distance highway group ranged between 50% - 98% across distance groups. The parcel geocoding probability for true exposure assignment ranged from 80% - 99%. The 0–50 m group had the lowest sensitivity for both street network geocoding datasets, and had the second lowest sensitivity for parcel geocoding.

**Table 2:** Distance bin misclassification by geocoding methodology

**Distance group (m)**	**# of Residences by proximity to highway**			**Measures of agreement**	
	**Tiger geocode**	**Orthophoto corrected**	**Confirmed match**	**Sensitivity**	**Specificity**
0–50	30	48	25	52.08%	99.17%
51–150	208	182	169	92.86%	91.61%
151–250	130	145	121	83.45%	98.21%
251–450	72	83	65	78.31%	98.76%
451– 999	30	10	8	80.00%	96.55%
>=1000	177	179	175	97.77%	99.57%
	**Streetmap USA**	**Orthophoto corrected**	**Confirmed match**	**Sensitivity**	**Specificity**
0–50	26	48	24	50.00%	99.67%
51–150	208	182	171	93.96%	92.04%
151–250	112	145	98	67.59%	97.21%
251–450	97	83	61	73.49%	93.62%
451–999	27	10	7	70.00%	96.86%
>=1000	177	179	176	98.32%	99.79%
	**Parcel geocode**	**Orthophoto corrected**	**Confirmed match**	**Sensitivity**	**Specificity**
0–50	41	48	40	83.33%	99.83%
51–150	192	182	180	98.90%	97.42%
151–250	141	145	134	92.41%	98.61%
251–450	86	83	80	96.39%	98.94%
451–999	10	10	8	80.00%	99.69%
>=1000	177	179	176	98.32%	99.79%

The analysis omits those addresses not successfully geocoded to TIGER, StreetMap USA and Parcel datasets (n = 647).

Confirmed match represents the number of residences classified in the distance group by each geocoding method and orthophoto corrected location assignment.

Sensitivity is the percentage of confirmed positive residences for each distance bin (confirmed match divided by orthophoto corrected).

Specificity is the percentage of confirmed negative residential locations for each distance bin (confirmed negative divided by the orthophoto corrected).

### Micro-environment time-activity

**Figure 4:**
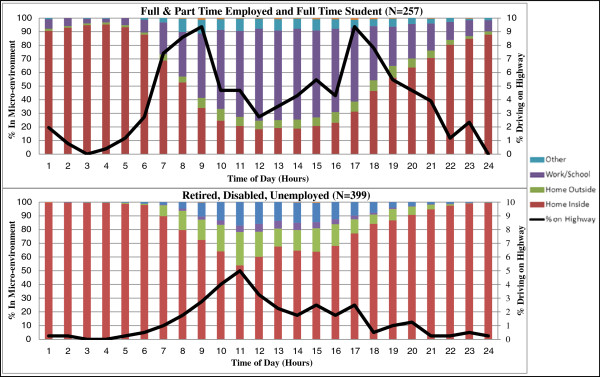
**Hourly micro**-**environment time**-**activity data for most recent workday**/**weekday by employment status.**

Hourly micro-environment time-activity patterns indicated differences in mobility patterns for workday/weekday and non-workday/weekend (Figure 4 & Additional file 2: Figure S1). Participants reporting full-time employment, part-time employment or being a full-time student had a diurnal pattern of travel that was significantly different from non-employed participants (Table 3 & Additional file 3: Table S2). The inside home micro-environment accounted for the largest workday/weekday and non-workday/weekend total time-activity allocation with a mean of 17.6 hrs and 19.4 hrs, respectively, for all participants (Table 3 & Additional file 3: Table S2). Micro-environment time-activity allocation was different for workday/weekday and non-workday/weekend (Table 3 & Additional file 3: Table S2). Hourly total time-activity patterns for the workday/weekday and non-workday/weekend of study participants differed significantly by age, race, employment status, educational attainment and income for the inside home and school/work micro-environments (Table 3 & Additional file 3: Table S2). Although there are significant differences in time-activity allocation for the five micro-environments only the differences in the inside home micro-environment consistently had a magnitude greater than one hour.

**Table 3:** **Work**-**day**/**Weekday micro**-**environment time**-**activity mean hours per day and percent of total 24**-**hour day**

	***Inside home***		***Outside home***		***School/work***		***Other***		***Highway***	
	**Mean (SD)**	**%/Day**	**Mean (SD)**	**%/Day**	**Mean (SD)**	**%/Day**	**Mean (SD)**	**%/Day**	**Mean (SD)**	**%/Day**
**All participants**	17.62 (4.4)	74%	1.61 (2.5)	7%	3.20 (4.5)	13%	1.26 (2.3)	5%	0.31 (0.9)	1%
**Gender**										
Male	17.22 (4.5)	72%	1.81 (2.8)	7%	3.35 (4.3)	14%	1.18 (2.1)	5%	0.44 (1.2)*	2%
Female	17.91 (4.3)	75%	1.45 (2.3)	6%	3.11 (4.5)	13%	1.32 (2.4)	5%	0.21 (0.5)*	<1%
**Age**										
< 60 yrs	15.85 (4.5)*	66%	1.42 (2.8)*	6%	5.09 (5.0)*	21%	1.27 (2.3)	5%	0.37 (0.7)*	2%
>= 60 yrs	19.48 (3.5)*	82%	1.81 (2.2)*	8%	1.22 (3.1)*	5%	1.25 (2.2)	5%	0.24 (1.0)*	1%
**Employment status**										
Full time working or student and part time working	13.75 (3.4)*	57%	1.12 (2.8)*	5%	7.54 (4.15)*	31%	1.05 (2.2)*	5%	0.54 (1.2)*	2%
Retired, disabled, homemaker or unemployed	20.09 (2.9)*	84%	1.92 (2.3)*	8%	0.42 (1.75)*	2%	1.41 (2.3)*	6%	0.16 (0.5)*	<1%
**Race**/**ethnicity**										
White	17.18 (4.4)*	72%	1.25 (2.4)*	5%	3.53 (4.5)	15%	1.71 (2.5)**	7%	0.33 (0.84)*	1%
Black	16.99 (4.6)	71%	0.86 (1.8)*	3%	3.51 (4.4)	15%	1.92 (2.9)**	8%	0.72 (1.9)***	3%
Asian	18.23 (4.2)*	76%	2.31 (2.6)**	10%	2.57 (4.3)	11%	0.73 (1.6)**	3%	0.16 (0.5)*	<1%
Other	18.07 (5.0)	76%	1.49 (2.6)	6%	3.57 (5.3)	15%	0.56 (1.4)**	2%	0.31 (0.5)*	1%
**Educational attainment**										
Less than high school diploma	18.45 (4.6)**	77%	2.19 (3.0)***	9%	2.22 (4.3)**	9%	0.97 (2.1)	4%	0.17 (0.5)	<1%
High school diploma	17.81 (4.2)*	74%	1.49 (2.4)*	6%	2.86 (4.4)*	12%	1.48 (2.6)	6%	0.36 (1.2)	2%
Undergraduate school	17.06 (4.38)*	71%	1.22 (2.0)*	5%	3.94 (4.7)*	16%	1.39 (2.1)	6%	0.39 (0.9)	2%
Graduate school	16.04 (3.9)**	67%	1.15 (2.1)*	5%	5.23 (4.6)**	22%	1.23 (2.0)	5%	0.35 (0.7)	1%
**Annual income**										
Less than $24,999	19.24 (3.73)**	80%	1.97 (2.6)**	8%	1.44 (3.4)**	6%	1.18 (2.3)	5%	0.17 (0.6)***	<1%
$25,000 – $74,999	15.82 (3.8)**	66%	1.3 (2.2)*	5%	4.91 (4.8)***	20%	1.58 (2.4)	7%	0.39 (0.7)*	2%
$75,000 or more	14.72 (4.0)**	61%	0.91 (1.9)*	4%	6.55 (4.6)***	27%	1.30 (2.3)	6%	0.52 (1.1)*	2%
Don’t know/ refused	18.26 (5.6)**	76%	1.64 (3.4)	7%	2.86 (5.03)**	12%	0.76 (1.6)	3%	0.48 (1.7)*	2%
**Study area**										
Somerville	17.14 (4.8)*	71%	0.95 (2.5)*	4%	3.95 (4.8)*	16%	1.56 (2.5)*	7%	0.4 (1.0)*	2%
South Boston/Dorchester	17.33 (4.4)*	72%	1.30 (2.3)*	5%	3.53 (4.6)*	15%	1.43 (2.4)*	6%	0.41 (1.1)*	2%
Chinatown	18.27 (4.10)**	76%	2.39 (2.6)**	10%	2.33 (4.2)**	10%	0.87 (1.8)**	3%	0.14 (0.4)**	<1%

Data is restricted to only those participants with a complete time-activity survey (N=663).

*, **, *** Indicates a significant mean difference from one or more group(s) within the same micro-environment.

Univariate regression models were developed to examine the association between workday time spent inside home and demographic variables including age, race, gender, educational attainment, income and employment status (Additional file 4: Table S3). The dichotomous variable employed/unemployed was found to be the largest predictor of total time spent inside the home (R^2^ = 0.49). A multi-variable regression model combining the demographic factors age, race, gender, educational attainment, income and employment status found that approximately 53% of the variability in total workday time spent inside home could be explained (Table 4). Race was not found to be a significant predictor in the multi-variable regression model and when removed the R^2^ was reduced to 52% (results not shown). A multi-variable regression model using the same demographic variables was developed to explain non-workday/weekend total hours spent in the inside home micro-environment, but was able to account for only 11% of the variability. Regression models also were run for the workday/weekday and non-workday/weekend second clinic visit and were found to have similar patterns (Table 4).

**Table 4:** **Mutli**-**variable regression models for total mean time spent inside home for workday**/**weekday and non**-**workday**/**weekend of 1**^**st **^**and 2**^**nd **^**survey**

	**Time activity survey 1 (N=652)**				**Time activity survey 2 (N=169)**			
	***Workday*/*weekday***		***Non*- *workday */*weekend***		***Workday*/*weekday***		***Non*- *workday */*weekend***	
	**R**^**2 **^**= 0.53**		**R**^**2\**^**= 0.11**		**R**^**2 **^**=0.48**		**R**^**2 **^**=0.16**	
	**ß**	**95% CI**	**ß**	**95% CI**	**ß**	**95% CI**	**ß**	**95% CI**
**Intercept**	12.65	(11.13, 14.19)	14.86	(13.18, 16.54)	12.47	(9.18, 15.77)	14.5	(10.32, 18.68)
**Age**	0.04	(0.02, 0.06)	0.04	(0.01, 0.06)	0.03	(−0.02, 0.08)	0.08	(0.01, 0.14)
**Male**	−0.40	(−0.88, 0.08)	0.04	(−0.49, 0.57)	−0.35	(−1.46, 0.75)	0.01	(−1.40, 1.41)
**Retired, disabled, or unemployed**	5.44	(4.84, 6.04)	0.65	(−0.01, 0.06)	4.59	(3.24, 5.93)	−0.35	(−2.06, 1.36)
**Education**								
Less than high school diploma	−1.19	(−2.15, −0.23)	0.18	(−0.88, 1.23)	−0.58	(−2.59, 1.42)	−0.54	(−3.08, 2.01)
High school diploma	−1.19	(−2.11, −0.27)	0.99	(−0.03, 2.00)	−0.56	(−2.22, 1.11)	0.01	(−2.1, 2.12)
Undergraduate School	−0.68	(−1.56, 0.20)	1.26	(0.30, 2.23)	−0.89	(−2.43, 0.64)	−1.23	(−3.18, 0.72)
Graduate School	Ref	Ref	Ref	Ref	Ref	Ref	Ref	Ref
**Income**								
Don’t know/ refused	0.90	(−0.19, 1.99)	0.53	(−0.67, 1.73)	0.53	(−1.96, 3.03)	1.6	(−2.48, 3.85)
Less than $24,999	1.46	(0.58, 2.35)	1.15	(0.18, 2.12)	2.91	(−0.49, 2.64)	1.79	(−0.54, 4.12)
$25,000 – $74,999	0.33	(−0.48, 1.14)	0.43	(−0.46, 1.32)	1.07	(−0.49, 2.64)	−0.24	(−2.22, 1.75)
$75,000 or more	Ref	Ref	Ref	Ref	Ref	Ref	Ref	Ref
**Race**								
White	−0.78	(−1.63, 0.06)	0.17	(−0.76, 1.10)	−0.61	(−2.42, 1.21)	−0.66	(−2.97, 1.64)
Black	−1.06	(−2.14, 0.02)	0.83	(−0.36, 2.01)	−0.76	(−2.97, 1.45)	−0.14	(−2.95, 2.67)
Asian	−0.85	(−1.70, −0.01)	0.87	(−0.05, 1.80)	0.42	(−2.29, 3.13)	3.44	(−0.01, 6.88)
Other	Ref	Ref	Ref	Ref	Ref	Ref	Ref	Ref

Analysis is restricted to those participants that had complete workday/weekday and non-workday/weekend questionnaires.

Workday/weekday micro-environment time-activity data indicated that a substantial portion of the population is not at home from 6–10 AM (Figure 4), a key exposure period when UFP levels are often elevated near the highway, including in the CAFEH study area in Somerville [5]. A logistic regression model was run to examine the odds of being inside of the home during each hour as a function of demographic variables (Additional file 5: Table S4). Employment status was significantly associated with the odds of being at home from 6–10 AM. Those with less than high school education were also significantly more likely to be inside the home during the 6–7 AM and 7–8 AM hours of the workday/weekday.

Time spent in each micro-environment was stratified by distance to highway bins to examine the relationship between proximity to highway and time-activity patterns. The 0–50 m group had the lowest mean time spent inside of the home for workday/weekday (16.3 hrs) and non-workday/weekend (18.4 hrs), and a significantly greater workday/weekday mean time spent at work/school (6.0 hrs) than all other distance groups. There was less variation in the time-activity patterns for non-workday/weekend by distance groups (Table 5).

**Table 5:** **Percent of population and mean time spent in microenvironments on workday/ weekday by distance to highway**

**Workday/weekday**										
	***Inside home***		***Outside home***		***School*/*work***		***Other***		***Highway***	
**Distance (m)**	**Mean (SD)**	**%/Day**	**Mean (SD)**	**%/Day**	**Mean (SD)**	**%/Day**	**Mean (SD)**	**%/Day**	**Mean (SD)**	**%/Day**
0–50	16.26 (5.0)*	67%	0.89 (1.6)	4%	5.98 (5.6)****	25%	0.64 (1.2)	3%	0.23 (0.4)	<1%
51–150	17.56 (4.2)	73%	1.90 (2.6)	8%	3.19 (4.5)*	13%	1.13 (2.1)	5%	0.22 (0.5)	<1%
151–250	18.39 (4.3)*	77%	1.57 (2.3)	6%	2.11 (3.9)**	9%	1.65 (2.9)	7%	0.28 (0.6)	1%
251–450	17.58 (4.3)	73%	1.75 (2.8)	7%	2.94 (4.2)*	13%	1.43 (2.3)	6%	0.30 (0.5)	1%
>=1000	17.48 (4.6)	73%	1.46 (2.7)	6%	3.46 (4.6)**	14%	1.18 (1.9)	5%	0.42 (1.39)	2%
**Non**–**workday/weekend**										
	***Inside home***		***Outside home***		***School/work***		***Other***		***Highway***	
**Distance (m)**	**Mean (SD)**	**%/Day**	**Mean (SD)**	**%/Day**	**Mean (SD)**	**%/Day**	**Mean (SD)**	**%/Day**	**Mean (SD)**	**%/Day**
0–50m	18.36 (4.2)*	77%	2.88 (4.5)	12%	1.06 (2.4)	4%	1.28 (2.1)	5%	0.42 (0.7)	2%
51–150m	19.37 (3.1)	81%	2.35 (2.6)	10%	0.48 (1.8)*	2%	1.61 (2.6)	6%	0.18 (0.4)	<1%
151–250m	20.22 (2.9)*	84%	2.05 (2.4)	9%	0.15 (1.0)*	<1%	1.41 (2.3)	6%	0.18 (0.4)	<1%
251–450m	19.52 (3.7)	81%	1.83 (2.5)	8%	0.35 (1.7)	2%	1.97 (2.6)	8%	0.33 (0.7)	1%
>=1000m	18.94 (4.1)*	79%	1.93 (2.9)	8%	1.18 (3.7)**	5%	1.67 (2.4)	7%	0.28 (0.6)	1%

*, **, **** Indicates a significant mean difference from one or more group(s) within the same micro-environment.

No significant mean difference in total time reported for workday/weekday micro-environment time-activity was detected between the first and second survey, and no micro-environment had a difference exceeding one hour (Table 6). Significant mean differences between first and second time-activity surveys were observed for the non-workday/weekend micro-environments school/work, other, and highway travel, but these differences constituted less than one hour.

**Table 6:** **Mean difference in total hours spent within each micro**-**environment between 1st and 2nd questionnaire** (**N**=**167**)

	**Micro**-**environment**	**Mean diff**	**95% CI**
**Workday/weekday (hrs)**	Inside home	0.09	(−0.48, 0.66)
	Outside home	−0.33	(−0.82, 0.15)
	School/work	0.48	(<−0.001, 0.96)
	Others	−0.24	(−0.81, 0.32)
	Highway	0.005	(−0.26, 0.15)
**Non**-**workday/weekend (hrs)**	Inside home	0.6	(−0.12, 1.31)
	Outside home	−0.02	(−0.59, 0.55)
	School/work	0.61	(0.15, 1.07)
	Others	−0.98	(−1.61, −0.29)
	Highway	−0.21	(−0.39, −0.04)

## Discussion

An exposure assignment that relies upon residential location with modest positional error may be appropriate for pollutants with less spatial and temporal variability than UFP (such as PM_2.5_). But for pollutants that decay rapidly as a function of distance from highways and major roadways, tens to hundreds of meters of positional error coupled with significant time spent away from home could have a profound effect on exposure misclassification, including possible differential misclassification.

### Geocoding positional error and exposure misclassification

Even when using validated geospatial databases, geocoding of addresses to street networks can introduce substantial positional error in studies where exposure may vary over tens of meters.. Our results indicate a median positional error of 22 m in our study domain using TIGER (Table 1), similar to Schootmann et al., who observed a median error of 31 m and 26 m when geocoding 261 residential addresses to an older version of TIGER and data from a commercial geocoding firm respectively [21]. Numerous other GIS studies have shown that geocoding addresses to the TIGER or commercially available street networks such as StreetMap can contribute larger amounts of positional error [10,19-21], but even the amount of spatial error in our study can potentially bias epidemiological results. This is highlighted by the lowest sensitivity in the highest exposure group (0–50 m proximity group), indicating that differential exposure misclassification related to geocoding was an issue for our population (Table 2).

The results of our analysis confirm the findings of a smaller sample (N = 126) by Zandbergen and Green, which identified a problem with sensitivity in the placement of school buildings into major roadway distance cut points when using street networks to geocode addresses [20]. Although the results of Zandbergen and Green may be affected by sample size, the distance cut-point groups they used (0–50 m, 51- 100 m and 101–150 m) led to sensitivities of 0%, 33% and 67% respectively for three out of four street-network geocoding databases [20]. Our sample size (N = 647) allowed for a more robust analysis of similar proximity cut points, but also found exposure misclassification to be differential with respect to distance to highway. Thus, effects of positional error on associations with health cannot be confidently assumed to be non-differential or biased only towards the null.

Geocoding to local or county created tax parcel databases is used less frequent in environmental health studies, but has been shown in this study and others to introduce less positional error than geocoding to street networks [19,23,26]. Parcel geocoding is considered and has been shown in previous literature to be more spatially accurate then street-network geocoding [19,21,23,26]. Our results deviate from previous literature and identify that parcel geocoding preformed no better than street-network geocoding when analysis was restricted to large multi-building parcels or large multi-unit buildings commonly seen in public and senior housing complexes (Table 1). We also discovered during the process of utilizing ortho-photos to ascertain true-ground location for large multi-unit parcels and buildings that these locations benefitted from manual adjustment that corrects the position to the physical location of the building through the use of scanned and georeferenced parcel/unit building layouts. Previous studies that have utilized parcel data sets have either not reported methodology for accurately assessing multi-building parcels or have restricted their analyses to remove these participants [19,26].

To the best of our knowledge, our efforts to reduce positional error through inclusion of ortho-photo and geo-referenced parcel/unit building layout maps in this study are the first application of these methods to environmental health research. The ability to do this was based partly on readily available datasets (MassGIS), pre-established relationships with housing authorities and our community partners reaching out to building management. This is an example of how CBPR can benefit the analytical aspect of environmental health research. We acknowledge that the manual steps taken to reduce positional error were conducive to the size of our study (N = 703). Researchers working with large cohorts will need to weigh the benefits of reducing positional error against the additional computational resources and time requirements. Conversely, smaller cohort studies that are able to reduce exposure misclassification from spatial error during the geocoding process by utilizing ortho-photo and scanned parcel/unit layouts will be able to increase their power to detect significant associations and reduce the need for larger cohorts. Researchers may want to at minimum geocode to parcel datasets, where available, to reduce positional error and consider restricting analysis by housing type. It may not be possible to completely eliminate positional error from the geocoding process, but recognizing the effects it can have on health analysis of pollutants with significant spatial variability is important.

### Micro-environment time-activity

Our study population spent the vast majority of its time within the inside-home microenvironment. As a result, personal exposure to ambient pollutants will depend on factors such as infiltration to the indoor residential environment, concentrations during time in traffic, and so forth. Studies using personal exposure monitoring and time-activity tracking through the use of Global Positioning System (GPS) units have shown that simple residentially assigned exposure to traffic-related pollutants misses significantly elevated exposures from time spent in or near heavy traffic [2,28]. Consequently, there is a need to develop exposure assessment models that integrate personal time-activity to more accurately assign exposure [11].

Our time-activity results indicated significant differences in mobility patterns of the study population across demographic variables as well as significant misclassification of exposure that is not equally distributed across all distance groups (Table 3 and Additional file 3: Table S2). Participants residing in the 0–50 m distance group reported significantly more hours at school/work on workday/weekday than all the other distance groups and spent the least amount of time within the home.

The relationship between proximity to highway and time-activity identified in this study introduces two issues epidemiologists may need to consider. First, models that assign ambient exposure to the residence may be a concern if there is meaningful geographic misclassification. Secondly, the fact that participants in the 0–50 m group spent more time at work in our sample suggests differential exposure to stressors in the workplace, as well as the possibility that this subgroup may differ by health status given their ability to work more hours (healthy worker effect). Exposure assessment models should consider adjustments for mobility patterns in their study populations and take into consideration inside home, on highway and work hour exposures to near-highway pollutants to reduce misclassification. Another approach would be to consider restricted analyses that separate employed and unemployed study participants.

People are highly mobile, potentially resulting in misclassification of exposure in studies that focus on pollutants with high spatial and temporal variability that do not consider non-residential exposures [15,16,29-31]. However, daily routines are relatively predictable, suggesting that only limited data are needed on a person’s activities to determine where they routinely spend their time [31]. Our results indicated that participants’ micro-environment activity patterns did not differ substantially even though the second questionnaire was completed in a different season than the first (Table 6). While anomalies in mobility patterns can be expected, the small amount of difference over time for time-allocation for individuals suggests modest data on time activity reasonably estimates yearly patterns. However, this may not be the case in longitudinal studies that follow individuals over a number of years since it has not been shown that mobility patterns remain constant year to year.

### Limitations and strengths

A strength of parcel geocoding was that it significantly reduced the amount of positional error for single family and small multi-family residences (i.e. duplexes and triple-deckers), but it was less successful at matching residential addresses than either StreetMap or TIGER. A previous study has shown that parcel address consistently had lower match rates when compared to geocoding to street-network or address point databases [23]. Most of the unmatched participants were from the South Boston study area and resided in a large multi-building public housing development. The inability to include these participants in the geocoding analysis may have biased the amount of positional error that occurs in large parcels with multiple buildings. Although our sample included random selection for a majority of the addresses, its generalizability is limited because the study areas are not necessarily representative of other near highway areas. Another limitation to our study was the modest number of participants that completed a full second micro-environment time-activity questionnaire for both a workday/weekday and non-workday/weekend (N = 167). This limited our ability to stratify further within the population to examine differences in repeatability along demographic variables.

A limitation of parcel geocoding is that it is typically generated by city or county planning or assessing departments and its availability in a GIS format conducive to geocoding can vary between cities. However, the localized generation of these data sources is also a strength since they are built with local knowledge which may result in a more updated address list than national street networks. Accessing these datasets will require effort for health researchers, including cross-discipline interactions with city planning departments in both academia and government.

Exposure misclassification due to geocoding has been limited to two-dimensions, with CAFEH study participants restricted to living four floors or fewer above ground level. We did not explore misclassification by vertical elevation which may contribute error if air pollutant exposure profiles are found to change as elevation increases. Further research is needed to develop new methods of location ascertainment that accommodate 3-dimensional building plans and to assess the accuracy before implementation in environmental health studies.

Physical activity data were collected on study participants (including duration, frequency, and intensity) but was not linked to the micro-environment time-activity data. Collection of this data would have allowed us to improve exposure misclassification further by including adjustments for rate of respiration. Future studies should consider incorporating physical activity into collection of time-activity data.

## Conclusions

We demonstrated that epidemiological studies focusing on proximity to major roadways could have reduced ability to detect true associations with adverse health effects due to inaccurate geocoding and the effects of population mobility. The magnitude of error related to geocoding practices was large relative to the steep concentration gradients of traffic-related air pollutants, and there was evidence of differential misclassification as a function of roadway proximity. In spite of this, to the best of our knowledge there are no health studies incorporating ortho-photo corrected geocoding efforts or reporting estimates of positional error when utilizing street-network geocoding. Time-activity patterns varied as a function of both roadway proximity and demographics, potentially complicating interpretation of multivariate epidemiological analyses. Future studies using increasingly sophisticated models of traffic-related air pollutants will require an emphasis on improving geocoding accuracy and integration of time-activity data. Geocoding and time-activity error are likely important in assessing health effects of exposure to near highway pollutants because these pollutants change rapidly in both space and time and people are moving in and out of the near highway environment.

## Abbreviations

ArcGIS: Geographic information system software; CAFEH: Community assessment of freeway exposure and health; CBPR: Community based participatory research; GIS: Geographic information system; Orthoimage: Aerial photograph that is geometrically corrected; TIGER: Topologically integrated geographic encoding and referencing; UFP: Ultrafine particles.

## Competing interests

Brugge has received travel support to make presentations about uranium mining from Friends of the Earth and International Physicians for the Prevention of Nuclear War.

## Authors’ contributions

KJL was the lead writer and analyst. MKS contributed to the writing and meaningful intellectual ideas to the analysis. JIL contributed to the writing and meaningful intellectual ideas that affected the interpretation and analysis of our data. CHF contributed to the literature review and provided intellectual ideas to the time-activity analysis, RP contributed to the analysis and development of figures for the time-activity data. WZ helped design the analysis and contributed to literature review. MM oversaw the statistical analysis and assisted in the writing of that section of the paper. DB directed the study, provided oversight to the analysis and contributed to the writing. All authors read the manuscript multiple times, provided input and approved the version as submitted.

## Supplementary Material

Additional file 1: Table S1 Descriptive statistics for distance in meters between each geocoding method and orthophoto corrected residential location by study areas. The analysis includes only those addresses successfully geocoded to all methods (n = 647). Click here for file

Additional file 2: Figure S1 Hourly micro-environment time-activity data for most recent workday/weekday and non-workday/weekend. Click here for file

Additional file 3: Table S2 Non-Workday/Weekend micro-environment time-activity mean hours per day and percent of total 24-hour day. Data is restricted to only those participants with a complete time-activity survey (N=663). Click here for file

Additional file 4: Table S3 Single regression models for workday/weekday hours spent inside home by demographic variables (N=653). Click here for file

Additional file 5: Table S4 Logistic regression of odds ratio for hourly inside home micro-environment during peak exposure window (N=653). Click here for file
