# Supplementary material for: Positional error and time-activity patterns in near-highway proximity studies: an exposure misclassification analysis
Source: Environ Health. 2013 Sep 8;12:75. doi: 10.1186/1476-069X-12-75 (PMC3907019; doi:10.1186/1476-069X-12-75)
Supplement: Additional file 1: Table S1 — Descriptive statistics for distance in meters between each geocoding method and orthophoto corrected residential location by study areas. The analysis includes only those addresses successfully geocoded to all methods (n = 647). [file 1476-069X-12-75-S1.docx]

**Supplemental table 1. Descriptive statistics for distance in meters between each geocoding method and orthophoto corrected residential location by study areas. The analysis includes only those addresses successfully geocoded to all methods (n = 647).**

|  | **Parcels** | **StreetMap USA** | **TIGER** |
| --- | --- | --- | --- |
| **Boston Neighborhoods** | | | |
| **Chinatown** |  |  |  |
| N | 156 | | |
| Mean (SD) | 32.93 (110)** | 43.63 (103.6)* | 54.29 (143)* |
| Median | 16.75 | 25.26 | 24.23 |
| 90^th^ Percentile | 71.65 | 78.43 | 92.65 |
| 95^th^ Percentile | 32.93 | 87.64 | 102.35 |
| Min - Max | 0-1352 | 4.59 - 1289 | 3.24 - 1288 |
| **Dorchester** |  |  |  |
| N | 201 | | |
| Mean (SD) | 12.67 (19.6)** | 29.29 (23.6)* | 27.42 (24.4)* |
| Median | 5.21 | 19.52 | 22.29 |
| 90^th^ Percentile | 32.21 | 58.32 | 61.97 |
| 95^th^ Percentile | 45.62 | 67.81 | 69.78 |
| Min - Max | 0.09 - 169 | 0.87 - 158 | 2.58 - 159.4 |
| **South Boston** |  |  |  |
| N | 14 | | |
| Mean (SD) | 53.37 (25.6)* | 39.82 (24.51) | 32.91 (21.3)* |
| Median | 53.21 | 41.41 | 23.1 |
| 90^th^ Percentile | 86.74 | 80.72 | 65.78 |
| 95^th^ Percentile | 114.85 | 86.06 | 79.41 |
| Min - Max | 18.1 - 114.9 | 9.7 - 86.1 | 11.3 - 79.4 |
| **All Boston Neighborhoods Combined** | |  |  |
| N | 371 | | |
| Mean (SD) | 22.78 (73.9)** | 35.74 (69.8)* | 38.99 (96)* |
| Median | 9.27 | 23.96 | 23.69 |
| 90^th^ Percentile | 50.92 | 67.81 | 72.67 |
| 95^th^ Percentile | 72.6 | 80.64 | 93.27 |
| Min - Max | 0 - 1352 | 0.8 - 1289 | 2.58 - 1288 |
| **Greater Boston Area** | | | |
| **Malden** |  |  |  |
| N | 78 | | |
| Mean (SD) | 13.59 (13.3)** | 30.52 (22.5)* | 42.37 (67)* |
| Median | 11.41 | 22.72 | 19.42 |
| 90^th^ Percentile | 34.6 | 34.6 | 76.65 |
| 95^th^ Percentile | 46.4 | 46.4 | 106.47 |
| Min - Max | 0 - 54.4 | 2.03 - 102.2 | 5.1 - 421.7 |
| **Somerville** |  |  |  |
| N | 198 | | |
| Mean (SD) | 22.25 (97.7)* | 47.54 (130.9)* | 70.30 (339.8) |
| Median | 6.18 | 20.12 | 19.37 |
| 90^th^ Percentile | 28.48 | 73.06 | 69.33 |
| 95^th^ Percentile | 44.49 | 94.03 | 105.61 |
| Min - Max | 0 - 1236.5 | 3.4 - 998.1 | 2.3 - 4453.3 |

*, **Indicates a significant mean difference from one or more geocoding method.
