# Supplementary material for: Positional error and time-activity patterns in near-highway proximity studies: an exposure misclassification analysis
Source: Environ Health. 2013 Sep 8;12:75. doi: 10.1186/1476-069X-12-75 (PMC3907019; doi:10.1186/1476-069X-12-75)
Supplement: Additional file 2: Figure S1 — Hourly micro-environment time-activity data for most recent workday/weekday and non-workday/weekend. [file 1476-069X-12-75-S2.docx]

**Supplemental Figure 1. Hourly micro-environment time-activity data for most recent workday/weekday and non-workday/weekend.**

**
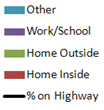
**
