# Supplementary material for: Positional error and time-activity patterns in near-highway proximity studies: an exposure misclassification analysis
Source: Environ Health. 2013 Sep 8;12:75. doi: 10.1186/1476-069X-12-75 (PMC3907019; doi:10.1186/1476-069X-12-75)
Supplement: Additional file 3: Table S2 — Non-Workday/Weekend micro-environment time-activity mean hours per day and percent of total 24-hour day. Data is restricted to only those participants with a complete time-activity survey (N=663). [file 1476-069X-12-75-S3.docx]

**Supplemental Table 2. Non-Workday/Weekend micro-environment time-activity mean hours per day and percent of total 24-hour day. Data is restricted to only those participants with a complete time-activity survey (N=663).**

|  | ***Inside Home*** | | ***Outside Home*** | | ***School/Work*** | | ***Other*** | | ***Highway*** | |
| --- | --- | --- | --- | --- | --- | --- | --- | --- | --- | --- |
|  | Mean (SD) | %/Day | Mean (SD) | %/Day | Mean (SD) | %/Day | Mean (SD) | %/Day | Mean (SD) | %/Day |
| **All Participants** | 19.42 (3.5) | 81% | 2.13 (2.8) | 8% | 0.61 (2.4) | 3% | 1.59 (2.4) | 7% | 0.25 (0.6) | 1% |
| **Gender** |  |  |  |  |  |  |  |  |  |  |
| Male | 19.41 (3.3) | 81% | 2.39 (2.9)* | 10% | 0.40 (1.7)* | 2% | 1.60 (2.5) | 7% | 0.20 (0.5) | <1 |
| Female | 19.42 (3.7) | 81% | 1.95 (2.6)* | 8% | 0.76 (2.8)* | 3% | 1.59 (2.4) | 7% | 0.28 (0.6) | 1% |
| **Age** |  |  |  |  |  |  |  |  |  |  |
| < 60 yrs | 18.77 (3.9)* | 78% | 2.32 (3.1) | 10% | 0.85 (2.77)* | 4% | 1.78 (2.5) | 7% | 0.28 (0.6) | 1% |
| >= 60 yrs | 20.09 (3.0)* | 84% | 1.94 (2.3) | 8% | 0.36 (1.89)* | 1% | 1.39 (2.35) | 6% | 0.22 (0.5) | <1% |
| **Employment Status** |  |  |  |  |  |  |  |  |  |  |
| Full time working or student and part time working | 18.44 (3.62)* | 77% | 2.30 (3.26) | 10% | 1.09 (2.85)* | 4% | 1.86 (2.49) | 8% | 0.31 (0.59)* | 1% |
| Retired, disabled, homemaker or unemployed | 20.02 (3.3)* | 84% | 2.02 (2.4) | 8% | 0.31 (2.02)* | 1% | 1.44 (2.4) | 6% | 0.21 (0.53)* | <1% |
| **Race/Ethnicity** |  |  |  |  |  |  |  |  |  |  |
| White | 18.96 (3.9)* | 79% | 1.80 (2.8)** | 7% | 0.87 (3.0)* | 4% | 2.04 (2.6)** | 9% | 0.33 (0.6)* | 1% |
| Black | 19.73 (3.1) | 82% | 1.17 (2.1)** | 5% | 0.68 (2.0) | 3% | 2.17 (2.7)* | 9% | 0.25 (0.5) | 1% |
| Asian | 20.09 (2.7)* | 84% | 2.55 (2.2)** | 11% | 0.20 (1.2)* | <1% | 1.01 (2.1)** | 4% | 0.14 (0.5)* | <1% |
| Other | 18.91 (4.3) | 79% | 2.96 (4.1)** | 12% | 0.80 (2.5) | 3% | 1.10 (2.2)* | 5% | 0.23 (0.5) | 1% |
| **Educational Attainment (Workday)** |  |  |  |  |  |  |  |  |  |  |
| Less than high school diploma | 19.42 (3.7)* | 81% | 2.73 (3.3)** | 11% | 0.28 (1.3)* | 1% | 1.37 (2.4)* | 6% | 0.20 (0.6) | <1% |
| High school diploma | 19.82 (3.1)* | 83% | 1.77 (2.2)* | 7% | 0.54 (2.0)* | 2% | 1.55 (2.5)* | 7% | 0.32 (0.6) | 1% |
| Undergraduate School | 19.61 (3.1)* | 82% | 1.94 (2.6)* | 8% | 0.77 (2.4) | 3% | 1.49 (2.2)* | 6% | 0.19 (0.4) | <1% |
| Graduate School | 17.83 (4.4)*** | 74% | 1.78 (2.5) | 8% | 1.43 (4.7)** | 6% | 2.61 (2.8)*** | 11% | 0.34 (0.6) | 1% |
| **Annual Income** |  |  |  |  |  |  |  |  |  |  |
| Less than $24,999 | 20.16 (2.9)** | 84% | 2.23 (2.5) | 9% | 0.26 (1.4)* | 1% | 1.16 (2.2)** | 5% | 0.19 (0.5)* | <1% |
| $25,000 – $74,999 | 18.75 (3.0)* | 78% | 2.00 (2.5) | 9% | 0.76 (2.2) | 3% | 2.20 (2.7)** | 9% | 0.29 (0.5) | 1% |
| $75,000 or more | 18.03 (4.1)* | 75% | 1.85 (2.6) | 8% | 1.45 (4.1)* | 6% | 2.31 (2.7)** | 10% | 0.36 (0.6)* | 1% |
| Don’t know/ refused | 19.35 (5.5) | 81% | 2.43 (4.7) | 10% | 0.79 (3.0) | 3% | 1.16 (2.2)** | 5% | 0.27 (0.6) | 1% |
| **Study Area** |  |  |  |  |  |  |  |  |  |  |
| Somerville | 18.56 (4.6)** | 77% | 1.72 (3.5)* | 7% | 1.46 (3.7)** | 6% | 1.89 (2.5)* | 8% | 0.37 (0.6)* | 2% |
| South Boston/Dorchester | 19.67 (3.3)* | 82% | 1.82 (2.4)* | 8% | 0.44 (1.9)* | 2% | 1.79 (2.5)* | 7% | 0.28 (0.5)* | 1% |
| Chinatown | 19.77 (2.8)* | 82% | 2.74 (2.34)** | 11% | 0.18 (1.2)* | <1% | 1.18 (2.3)** | 5% | 0.13 (0.5)** | <1% |
| *, **, ***, **** Indicates a significant mean difference from one or more group(s) within the same micro-environment. | | | | | | | | | | |
