# Supplementary material for: Positional error and time-activity patterns in near-highway proximity studies: an exposure misclassification analysis
Source: Environ Health. 2013 Sep 8;12:75. doi: 10.1186/1476-069X-12-75 (PMC3907019; doi:10.1186/1476-069X-12-75)
Supplement: Additional file 4: Table S3 — Single regression models for workday/weekday hours spent inside home by demographic variables (N=653). [file 1476-069X-12-75-S4.docx]

**Supplemental Table 3. Single regression models for workday/weekday hours spent inside home by demographic variables (N=653).**

|  | **Hours** | **95%CI** | **R^2^** |
| --- | --- | --- | --- |
| **Age** | 0.16 | 0.13, 0.18 | 0.21 |
| **Male** | -0.69 | -1.38, -0.02 | 0.01 |
| **Retired, disabled, or unemployed** | 6.34 | 5.84, 6.83 | 0.49 |
| **Race** |  |  |  |
| White | -0.89 | -2.01, 0.24 | 0.01 |
| Black | -1.07 | -2.58, 0.43 | -- |
| Asian | 0.16 | -0.99, 1.32 | -- |
| Other | Ref | Ref | -- |
| **Education** |  |  |  |
| Less than high school diploma | 2.41 | 1.25, 3.57 | 0.03 |
| High school diploma | 1.77 | 0.59, 2.94 | -- |
| Undergraduate School | 1.01 | -0.18, 2.20 | -- |
| Graduate School | Ref | Ref | -- |
| **Income** |  |  |  |
| Don’t know/ refused | 3.54 | 2.26, 4.82 | 0.18 |
| Less than $24,999 | 4.52 | 3.62, 5.42 | -- |
| $25,000 – $74,999 | 1.10 | 0.10, 2.10 | -- |
| $75,000 or more | Ref | Ref | -- |
| **Study Area** |  |  |  |
| Somerville | -1.12 | -1.98, -0.27 | 0.01 |
| Dorchester/South Boston | -0.94 | -1.72, -0.16 | -- |
| Chinatown | Ref | Ref | -- |
