# Supplementary material for: Positional error and time-activity patterns in near-highway proximity studies: an exposure misclassification analysis
Source: Environ Health. 2013 Sep 8;12:75. doi: 10.1186/1476-069X-12-75 (PMC3907019; doi:10.1186/1476-069X-12-75)
Supplement: Additional file 5: Table S4 — Logistic regression of odds ratio for hourly inside home micro-environment during peak exposure window (N=653). [file 1476-069X-12-75-S5.docx]

**Supplemental Table 4. Logistic regression of odds ratio for hourly inside home micro-environment during peak exposure window (N=653).**

|  | ***6 - 7 AM*** | | ***7 - 8 AM*** | | ***8 - 9 AM*** | | ***9 - 10AM*** | |
| --- | --- | --- | --- | --- | --- | --- | --- | --- |
|  | R^2^ = 0.09 | | R^2^= 0.11 | | R^2^ =0.18 | | R^2^ =0.20 | |
|  | OR | 95% CI | OR | 95% CI | OR | 95% CI | OR | 95% CI |
| **Age (>=60yrs old)** | 1.02 | (1.0, 1.04) | 1.01 | (0.99, 1.03) | 1.00 | (0.98, 1.01) | 0.98 | (0.97, 1.00) |
| **Male** | 1.11 | (0.73, 1.69) | 0.97 | (0.68, 1.39) | 1.27 | (0.90, 1.80) | 0.89 | (0.62, 1.26) |
| **Full & Part Time Work, Student** | 0.19 | (0.11, 0.33) | 0.30 | (0.19, 0.46) | 0.30 | (0.20, 0.45) | 0.30 | (0.20, 0.46) |
| **Education** |  |  |  |  |  |  |  |  |
| Less than high school diploma | 2.64 | (1.16, 6.03) | 2.99 | (1.44, 6.24) | 0.73 | (0.36, 1.48) | 0.67 | (0.32, 1.39) |
| High school diploma | 1.59 | (0.72, 3.52) | 1.83 | (0.91, 3.68) | 0.81 | (0.42, 1.59) | 0.95 | (0.47, 1.93) |
| Undergraduate School | 1.37 | (0.65, 2.87) | 1.79 | (0.93, 3.43) | 0.87 | (0.45, 1.65) | 0.86 | (0.43, 1.72) |
| Graduate School | Ref | Ref | Ref | Ref | Ref | Ref | Ref | Ref |
| **Income** |  |  |  |  |  |  |  |  |
| Don’t know/ refused | 0.87 | (0.35, 2.18) | 0.28 | (0.12, 0.65) | 0.36 | (0.17, 0.80) | 0.46 | (0.21, 1.01) |
| Less than $24,999 | 0.72 | (0.34, 1.52) | 0.31 | (0.16, 0.59) | 0.81 | (0.42, 1.59) | 0.51 | (0.27, 0.97) |
| $25,000 – $74,999 | 1.04 | (0.55, 1.95) | 0.70 | (0.40, 1.23) | 0.87 | (0.45, 1.65) | 1.11 | (0.59, 2.08) |
| $75,000 or more | Ref | Ref | Ref | Ref | Ref | Ref | Ref | Ref |
| **Race** |  |  |  |  |  |  |  |  |
| White | 1.11 | (0.53, 2.33) | 0.55 | (0.29, 1.03) | 1.08 | (0.58, 2.00) | 1.29 | (0.69, 2.40) |
| Black | 1.56 | (0.65, 3.91) | 0.92 | (0.42, 2.02) | 1.46 | (0.66, 3.21) | 1.16 | (0.52, 2.57) |
| Asian | 0.79 | (0.37, 1.68) | 0.78 | (0.42, 1.44) | 0.95 | (0.51, 1.77) | 1.06 | (0.57, 1.97) |
| Other | Ref | Ref | Ref | Ref | Ref | Ref | Ref | Ref |
